# Perceptions of digital medical information services applying new technologies

**DOI:** 10.5195/jmla.2026.2314

**Published:** 2026-07-01

**Authors:** Go Eun Lee, JungSun Seo, Ye Jin Choi, Jee Yeon Lee

**Affiliations:** 1 ggoni3530@yonsei.ac.kr, Researcher, Research Institute for Academic Library Development, Yonsei University, Seoul, South Korea; 2 seoooo0316@yonsei.ac.kr, Doctoral Student, Department of Library and Information Science, Yonsei University, Seoul, South Korea; 3 brightyejin@daegu.ac.kr, Assistant Professor, Department of Library and Information Science, Daegu University, Gyeongsan, South Korea; 4 jlee01@yonsei.ac.kr, Professor, Department of Library and Information Science, Yonsei University, Seoul, South Korea

**Keywords:** Digital Health information, Medical Libraries, Emerging Technologies, Artificial Intelligence, Information Quality, User Satisfaction

## Abstract

**Objectives:**

This study examines users& behaviors and perceptions when accessing medical information in digital environments and proposes strategic directions for medical libraries seeking to adopt emerging technologies. Specifically, we empirically investigate user expectations regarding digital technologies, differences in perception based on individual characteristics, and key factors influencing satisfaction with information use.

**Methods:**

We conducted a web-based survey from January to February 2024 through the National Center for Medical Science Knowledge, a national institution in South Korea. A total of 580 participants—including healthcare professionals, researchers, educators, and students—completed a questionnaire assessing digital information behavior, preferences for emerging technologies, perceptions of information and service quality, and overall satisfaction. We analyzed the data using descriptive statistics, analysis of variance (ANOVA), and multiple regression analysis.

**Results:**

Users exhibited a strong tendency to use online-based medical information, reporting particular interest in big data (38.5%) and artificial intelligence (24.9%) technologies. Statistically significant differences in preferences and awareness of digital technologies were observed based on gender, education level, academic major, and occupation. Information reliability (β = .623), service speed, and personalization were identified as key determinants of user satisfaction.

**Conclusion:**

Medical libraries should establish technology-based services that are suitable for the digital information environment and develop customized strategies that accurately reflect user characteristics and demands. This study offers practical insights for designing user-centered digital medical information services.

## INTRODUCTION

Recent advances in emerging technologies—such as artificial intelligence (AI), big data, cloud computing, and the Internet of Things (IoT)—have fundamentally transformed the creation, distribution, and utilization of medical information. These technologies enhance accessibility and enable the delivery of personalized healthcare services, but they also introduce new challenges related to technology acceptance and digital disparities [1]. In the medical domain, where timely and accurate information can be a matter of life and death, building trustworthy digital health information systems is becoming a priority [2]. Medical libraries have long been recognized as institutions that provide access to specialized knowledge and professional services, and their role has been further expanded and reinforced by recent advances in digital technologies [3].

In response to these technological advancements and the increasing expectations of users, medical information service providers must prioritize the expansion of digital content and the development of user-centered, personalized search services [4]. Previous studies suggest that users increasingly expect technology-enabled services—not merely in terms of digitalization, but in leveraging intelligent data systems and personalized service experiences [5]. These trends indicate a broader shift in the medical information environment, from basic information dissemination toward real-time data analysis and tailored information solutions.

Against this backdrop, this study aims to systematically examine how users perceive emerging digital technologies and which aspects of these technologies shape their expectations of medical information. The findings seek to inform future strategies for medical information services by foregrounding the user perspective and identifying practical approaches to building an effective digital ecosystem.

In this context, understanding the scope of medical information handled by medical libraries is essential. The types of information generated in the field of medical science range from scholarly literature to experimental results, genomic data, and disease statistics [6]. Notably, since the COVID-19 pandemic, a diverse range of users—from medical professionals to patients and the general public—have shown increasing interest in medical information [7]. In general, as user groups diversify, their information needs and behaviors begin to vary significantly depending on their experiences, environments, and purposes. For instance, healthcare professionals demand timely and accurate information to ensure a high quality of care[8], whereas patients tend to prefer intuitive and easily accessible information when seeking to understand and manage their health[9]. Given these diverse needs, medical libraries must adopt new strategies in implementing digital technologies and designing services. As a crucial step, determining whether such technologies genuinely benefit users and how the quality of information and services impacts satisfaction can enable libraries to develop effective service models that are based on the effective integration of digital technologies.

Recent studies document concrete implementations of digital technologies in medical libraries, including AI-assisted search integrated in library-managed information services, cloud-based environments, automated interlibrary loan and document-delivery platforms, mobile reservation interfaces, and text-mining tools designed to support clinical information use [10–12]. In this context, digital medical information encompasses both the medical content itself and the digital environments and platforms through which it is accessed and utilized [13,14].

Prior work has identified key information-related factors shaping users’ satisfaction with digital medical information, including topical relevance, source credibility, and recency. In addition, studies have reported technology- and system-related barriers, such as time constraints, access limitations, and search complexity, which can hinder effective information use and indirectly influence user satisfaction [15–17]. Together, these findings indicate that, while digital technologies can enhance information quality and accessibility, they may also introduce new challenges to effective information use. Accordingly, medical libraries need to balance the adoption of emerging technologies with continuous evaluation of how such innovations impact user satisfaction and service quality.

With these points in mind, this study analyzes perceptions among users of medical science information regarding the use of digital medical information and the adoption of new technologies, with the ultimate goal of devising practical insights for medical libraries in designing customized services that are appropriate for digital environments. By examining differences in information usage and technology awareness based on factors such as gender, age, education, and occupation and identifying preferred digital technology elements, we hope to offer guidance for improving public medical information services in the era of digital transformation. We expect that our findings will contribute to the academic development of information science and medical librarianship and serve as foundational data for designing user-centered, customized services.

Thus, our research questions are as follows:

1. What are the digital information usage behaviors of medical information users?
2. What digital technologies would users like to see implemented in medical libraries?
3. Are there differences in technology perceptions depending on users’ personal characteristics?
4. What factors influence satisfaction with the use of digital medical information?

## METHODS

This study aims to propose directions for developing information services in medical libraries through the integration of digital technologies. First, we examined the literature on information services and digital technology integration in medical libraries and identified applicable technologies and services. Then, we designed and administered a survey to medical information users to explore user preferences for digital technologies and platforms.

To systematically examine users’ perceptions and expectations of emerging technologies, it is first necessary to establish a clear and consistent classification framework. Accordingly, this study adopts the technology types defined in the Classification System for Fourth Industrial Revolution Technologies developed by the Korean Intellectual Property Office (KIPO), which categorizes emerging technologies into six types: artificial intelligence (AI), big data, cloud computing, the Internet of Things (IoT), next-generation mobile communication, and virtual/augmented reality (VR/AR). For this study, IoT and next-generation mobile communication were merged due to their functional similarities, resulting in five consolidated domains.

Based on this framework, we reviewed the existing cases and literature on the application of digital technologies in libraries and categorized them into five types: (1) virtual/augmented reality, (2) big data, (3) artificial intelligence, (4) next-generation/mobile communication and IoT, and (5) cloud computing [18,19]. To clarify these categories, this study defines them with illustrative examples. Artificial intelligence refers to services like AI-based information retrieval and summarization systems (e.g., ChatGPT, Gemini). Big data analytics includes platforms for analyzing user data and trends (e.g., Tableau, R-based analysis tools). Cloud computing encompasses remote access platforms (e.g., AWS, Google Cloud), while next-generation mobile technologies include services like 5G-based mobile library applications. Finally, virtual/augmented reality refers to immersive learning environments (e.g., VR-based medical training simulations). These five technology domains served as the analytical framework for examining medical and health information users’ perceptions and expectations regarding the application of emerging technologies in future services.

### Survey Development

To help respondents better understand the scope and potential application of each technology domain, we included examples of how each might be used in medical library contexts. Specifically, the questionnaire proposed the use of virtual/augmented reality for user education and information literacy programs; next-generation/mobile/IoT technologies for services related to external resource access and interlinking; and cloud computing for providing online services, remote access to electronic materials, and E-learning systems. These use-case examples were presented alongside the corresponding technology category to guide participant responses and elicit targeted feedback.

In designing the questionnaire, this study drew upon a comprehensive review of domestic and international environmental changes in medical information services, documented cases of technology applications in medical libraries, and prior research literature on user satisfaction and digital library systems. Building on these empirical insights, the survey was theoretically grounded in the Information System (IS) Success Model proposed by DeLone and McLean (2003). This model conceptualizes the performance of information systems through the dimensions of information quality, system quality, service quality, and user satisfaction. Among these, the present study focused on information and service quality as the primary predictors of satisfaction with digital medical information, given the context of digital library services where users predominantly engage with content and interfaces rather than backend infrastructure. Accordingly, system quality was considered less central and was not included in the core analysis. Questions on information quality addressed the accuracy and relevance of digital content, while those on service quality captured ease of use, accessibility, and responsiveness. The survey comprised two sections on general use and digital transformation technologies. The former assessed user perceptions of experience, services, programs and the latter included questions on understanding and proficiency regarding digital technologies, desired services, perceptions of copyright and privacy protection regarding digital resources, and preferences for digitally integrated services.

The questionnaire included a mix of five-point Likert scale items, open-ended questions, and multiple-response items. A pilot test was conducted with nine participants, and the survey was refined accordingly.

### Survey dissemination

The web-based survey was conducted from January 26 to February 16, 2024, targeting users of medical and health-science information services. To ensure broad participation, the study was conducted in collaboration with multiple national-level institutions that serve as major providers of medical and health-science information in South Korea, including the National Center for Medical Science Knowledge (NCMK), the National Institute of Health, and the Korean Medical Library Association. Participants included professionals from diverse fields who had experience using medical and health information, such as physicians, researchers, educators, students, and nurses. Survey invitations were distributed through the official websites, newsletters, affiliated medical libraries, professional mailing lists, and social-media channels of these institutions. Each cooperating institution announced the study on its website, describing the research purpose and significance and providing a link to the online questionnaire. Users voluntarily accessed the survey link and participated anonymously. No personal identifiers were collected, and participation was entirely voluntary.

This study was reviewed using the IRB self-assessment checklist of the authors' affiliated institution and determined to be exempt from full IRB review pursuant to Article 15, Paragraph 2 of the Bioethics and Safety Act of the Republic of Korea and Article 13 of its Enforcement Rules, which exempt human subjects research that utilizes publicly available information or that does not collect or record personally identifiable information about

Finally, we performed multiple regression analysis to identify data and service quality factors influencing satisfaction with digital medical information. All statistical analyses were conducted using IBM SPSS Statistics (version 26). Survey responses were research participants. Formal confirmation of exemption was issued by the relevant administrative office of the authors' affiliated institution.

### Data Analysis

We performed a frequency analysis to examine the distribution of the data regarding demographics, information behavior, and the use of digital technologies. To examine differences in perceptions of emerging technologies based on users’ personal characteristics, we conducted chi-square tests, which are appropriate for analyzing relationships between categorical variables. exported from Google Forms and initially cleaned in Microsoft Excel by removing incomplete entries and recoding categorical variables. The refined dataset was then imported into SPSS for further analysis.

Data and materials are available in OSF at https://osf.io/3b6jh/files/osfstorage.

**Figure 1:**
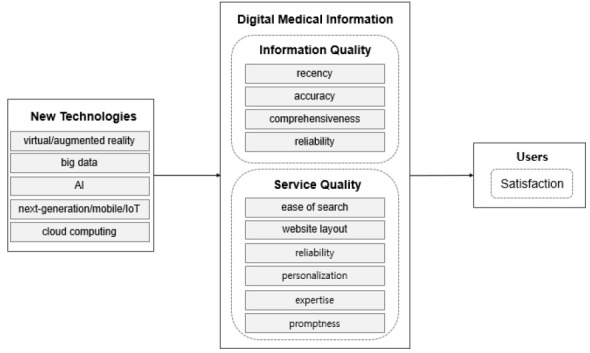
Relationship between Emerging Technologies, Digital Medical Information, and Information Use Satisfaction

## RESULTS

### 1. Demographic Characteristics

Of the 586 collected responses, 580 were included in the final analysis after excluding incomplete or inattentive responses. The sample consisted primarily of female participants, individuals in their 30s and 40s, and respondents holding graduate-level degrees. Most participants were healthcare professionals or researchers, with less than 15 years of professional experience. Table 1 presents the demographic characteristics of the respondents.

**Table 1:** Demographic characteristics of respondents (N = 580)

Variable	Category	n	Percentage
Gender	Male	215	37.1
	Female	365	62.9
Age	Teens	3	0.5
	20s	83	14.3
	30s	186	32.1
	40s	201	34.7
	50s	94	16.2
	60s and older	13	2.2
Education	High school	6	1.0
	Junior college	31	5.3
	University	190	32.8
	Graduate school	351	60.5
	Other	2	0.3
Major	Medical fields	271	46.7
	Science/Engineering	143	24.6
	Humanities/Social sciences	121	20.9
	Other	45	7.8
Occupation of Education	Healthcare	30	11.1
	Research	32	11.8
	Medical Majors	99	36.5
	Student	96	35.4
	Other	14	5.2
Work experience	< 2 years	60	10.3
	2-5 years	86	14.8
	5-10 years	118	20.4
	10-15 years	105	18.1
	15-20 years	83	14.3
	20-25 years	69	11.8
	≥ 25 years	57	9.8

Note: Occupation was assessed only among respondents who identified their major as medical-related (n=271).

### 2. Perceptions of the Use of Medical Information and Technology

- **Usage Patterns of Digital Resources**

When asked how they primarily access medical information, 59.8% (347/580) of respondents reported using online (website-based) services, while 40.2% (233/580) relied more on offline (in-person) methods. This suggests that medical information users primarily rely on electronic resources, databases, and information sources. Regarding the most frequently referenced types of medical information resources, scholarly articles in medical science (45%, 261/580) and research outputs from medical research institutions or projects, such as internal reports, datasets, or other unpublished research deliverables (22.9%, 133/580) comprised the majority, while general health-related information (19%, 110/580) and publications by government agencies or ministries (10.7%, 68/580) were used less frequently.

Frequently used platforms in the medical and scientific fields indicated a wide range of platforms, including Access Medicine, national information agencies (e.g., National Library of Korea, National Assembly Library), ProQuest Medical Library, JCR (Journal Citation Reports), university library services (e.g., library websites, discovery systems, and institutional repositories), the Korea Institute of Science and Technology Information (KISTI), Wiley Online Library, and the Korea Education and Research Information Service (KERIS). Among these, KISTI is an academic information portal for Korean researchers in science, technology, and medicine, and KERIS supports the joint utilization of academic resources across university libraries.

Although users rated the importance of medical information resources very highly (average (mean) score = 4.6), their perception of the usefulness of the resources provided by medical libraries (3.96) and satisfaction with digital services and electronic resources (3.81) were comparatively lower.

- **Perceptions of Digital Technologies and Services**

When asked about the perceived advantages of using emerging digital technologies in medical library services, users most frequently selected the following advantages: summarized information (24.8%, 144/580 respondents), ease of access (21.0%, 122/580), prompt information acquisition (19.5%, 113/580), personalized information (18.3%, 106/580), ease of tracking current trends (13.6%, 79/580), and trust in the acquired information (2.8%, 16/580). There is a high demand for practical, technology-oriented training. Respondents selected the following: how to use analytical and research assistance tools (186/580, 32.1%), guide to collection and database usage (24.1%, 140/580), guide to the latest AI technology (22.4%, 130/580), information on how to search for medical science information (12.1%, 70/580), library user guide for accessing and using digital services (8.6%, 50/580), and others (0.7%, 4/580).

Indeed, despite users recognizing the value of digital technologies, their self-rated levels of understanding (3.14) and proficiency (3.03) were relatively low.

Regarding the types of digital technologies users wish to apply in research and clinical settings, the most desired were big data (38.5%, 223/580) and AI (24.9%, 144/580), followed by cloud computing (11.5%, 67/580), next-generation/mobile communication (8.9%, 52/580), virtual and augmented reality (8.2%, 48/580), IoT (7.0%, 41/580), and other technologies (0.9%, 5/580). Based on this classification, we also identified areas within library services that can adopt these technologies and matched them accordingly (see Table 2).

**Table 2:** Integration of Digital Technologies and Specific Library Services

Type	Service	No. of responses	Percentage	Rank
Virtual/augmented reality	Technology-Integrated Creative Learning Spaces	287	46.3	2
	Information Literacy/User Education	333	53.7	1
Big data	Trend/Network Analysis	216	18.4	2
	Multimedia Resource Provision/Guidance	100	8.5	5
	Provision/Use of Library Holdings	135	11.5	3
	Research Capacity Enhancement Support	270	23.0	1
	Online Service Provision/Guidance	90	7.5	7
	E-learning Provision/Integration	131	11.2	4
	Information Literacy/User Education	82	7.0	8
	Knowledge & Information Curation	51	4.2	9
	Academic Journal Information Guidance	97	8.3	6
Artificial intelligence	Research Capacity Enhancement Support	351	39.9	1
	Research Ethics Guidance/Plagiarism Prevention	148	16.8	3
	Online Service Provision/Guidance	162	18.4	2
	Summarization Service	123	14.0	4
	Information Literacy/User Education	96	10.9	5
Next-generation/mobile communication/IoT	Personal Research Space Reservation	166	13.2	1
	Group Activity Space Reservation	109	8.7	6
	Multimedia Resource Provision/Guidance	94	7.5	8
	Center Access Control	49	3.9	11
	Provision/Use of Library Holdings	124	9.8	5
	Technology-Integrated Creative Learning Spaces	125	9.9	4
	Online Service Provision/Guidance	92	7.3	9
	External Resource Provision/Integration	161	12.8	2
	Remote Access to Electronic Resources	151	12.0	3
	E-learning Provision/Integration	99	7.9	7
	Information Literacy/User Education	56	4.4	10
	Knowledge & Information Curation	39	3.1	12
Cloud computing	Online Service Provision/Guidance	280	36.1	1
	Remote Access to Electronic Resources	230	29.7	3
	E-learning Provision/Integration	265	34.2	2

Note: Participants were allowed to select more than one option. Definitions and detailed descriptions of each service category are provided in the codebook in Appendix B.

Next, we examined how users who wanted to utilize these technology-enhanced services perceived the quality of information (recency, accuracy, comprehensiveness, reliability) and services (ease of search, website layout, reliability, promptness, personalization, expertise) in the context of digital medical information. As shown in Table 3, services based on AI, VR/AR, big data, cloud, mobile, and IoT technologies received relatively high evaluations for both (information) reliability and (service) expertise, with mean scores greater than 4.1 indicating that users expect new technologies to serve as core tools for delivering accurate, professional digital library services.

**Table 3:** Perceptions of Information and Service Quality in Digital Medical Information by Type of Technology-Based Library Services (N=580)

Type	Service	Information quality		Service quality							
		Recency	Accuracy	Comprehensiveness	Reliability	Ease of search	Website layout	Reliability	Promptness	Personalization	Expertise
Virtual/augmented reality	Technology-Integrated Creative Learning Spaces	3.70	3.90	3.72	4.07	3.59	3.61	4.02	3.70	3.38	3.87
	Information literacy/user education	3.76	3.96	3.79	4.17	3.73	3.76	4.14	3.82	3.64	3.96
Big data	Trend/network analysis	3.76	3.94	3.72	4.15	3.63	3.57	4.08	3.75	3.43	3.94
	Multimedia resource provision/guidance	3.65	3.89	3.70	4.07	3.51	3.53	4.06	3.67	3.32	3.87
	Provision/use of library holdings	3.69	3.90	3.72	4.07	3.59	3.60	4.02	3.70	3.39	3.87
	Research capacity enhancement support	3.64	3.90	3.73	4.10	3.52	3.56	4.01	3.68	3.37	3.84
	Online service provision/guidance	3.63	3.97	3.62	4.09	3.46	3.48	3.96	3.56	3.22	3.89
	E-learning provision/integration	3.63	3.99	3.7	4.15	3.49	3.75	4.12	3.75	3.25	3.99
	Information literacy/user education	3.68	3.95	3.77	4.14	3.57	3.69	4.1	3.75	3.46	3.93
	Knowledge & information curation	3.71	3.97	3.77	4.16	3.61	3.64	4.08	3.73	3.37	3.91
	Academic journal information guidance	3.84	4.03	3.78	4.13	3.72	3.61	4.12	3.77	3.46	3.97
Artificial intelligence	Research capacity enhancement support	3.7	3.86	3.72	4.09	3.58	3.6	3.97	3.69	3.4	3.84
	Research ethics guidance/plagiarism prevention	3.72	4.01	3.76	4.19	3.56	3.65	4.14	3.74	3.36	4.02
	Online service provision/guidance	3.65	3.89	3.65	4.11	3.59	3.58	4.03	3.74	3.35	3.84
	Summarization service	3.74	3.97	3.72	4.07	3.60	3.63	4.01	3.71	3.43	3.89
	Information literacy/user	3.64	3.91	3.71	3.99	3.58	3.55	3.96	3.63	3.4	3.83
Next-generation· Mobile communication/·IoT	Personal research space reservation	3.67	3.89	3.63	4.06	3.57	3.59	3.99	3.62	3.31	3.83
	Group activity space reservation	3.68	4.00	3.64	4.08	3.56	3.66	4.10	3.62	3.42	3.94
	Multimedia resource provision/guidance	3.63	3.92	3.71	4.14	3.56	3.57	4.08	3.70	3.31	3.90
	Center access control	3.61	3.82	3.66	4.15	3.55	3.55	4.03	3.63	3.32	3.90
	Provision/use of library holdings	3.68	3.88	3.76	4.13	3.65	3.61	4.09	3.71	3.39	3.89
	Technology-Integrated Creative Learning Spaces	3.76	4.08	3.85	4.25	3.67	3.71	4.18	3.73	3.32	3.95
	Online service provision/guidance	3.76	4.10	3.76	4.22	3.55	3.58	4.15	3.66	3.28	3.95
	External resource provision/integration	3.70	3.93	3.64	4.16	3.56	3.53	4.11	3.70	3.33	3.93
	Remote access to electronic resources	3.68	3.86	3.59	4.04	3.57	3.56	3.99	3.68	3.37	3.83
	E-learning provision/integration	3.54	3.81	3.75	4.04	3.42	3.58	4.00	3.75	3.33	3.84
	Information literacy/user education	3.73	3.94	3.78	4.09	3.69	3.66	4.01	3.75	3.45	3.90
	Knowledge & information curation	3.71	4.02	3.75	4.16	3.50	3.52	4.12	3.70	3.28	3.92
Cloud computing	Online service provision/guidance	3.71	3.88	3.71	4.09	3.61	3.66	4.03	3.75	3.40	3.90
	Remote access to electronic resources	3.74	3.94	3.79	4.13	3.79	3.68	4.11	3.81	3.55	3.85
	E-learning provision/integration	3.51	3.59	3.45	3.75	3.39	3.43	3.63	3.47	3.27	3.63

Note: Responses were measured using a 5-point Likert scale (1 = *strongly disagree* to 5 = *strongly agree*). Values represent mean scores.

### 3. Differences in Technology Perception by Personal Characteristics

We conducted a chi-square test to determine whether perceptions of emerging technologies differ according to users’ personal characteristics. Gender had a significant effect on users’ perceived understanding of and proficiency with digital technologies. Specifically, female respondents rated their understanding (χ² = 15.070, df = 4, p < .01) and proficiency (χ² = 19.326, df = 4, p < .01) lower than males. This suggests that women may feel less confident or less familiar with technologies such as computer-based tools or systems and artificial intelligence.

The types of technologies users hoped to see integrated also differed depending on their educational background (χ² = 65.999, df = 16, p < .01). While graduate-level respondents expressed strong interest in the application of cloud computing, big data, and virtual/augmented reality in medical information services, university graduates reported strong interest in next-generation and mobile communication technologies. Junior college graduates notably favored the IoT, while high school graduates expressed high interest in AI, IoT, and cloud computing. Additional responses under “Other” included demands for technologies related to statistics or experimental applications. Taken together, these findings suggest that technology preferences and expectations differ based on educational attainment.

Users’ preferred types of technologies for medical information use also varied depending on their academic major (χ² = 30.388, p < .01). Respondents in medical-related fields reported the highest interest across all technology types, particularly in AI and cloud-based services, and strongly preferred services related to online access and guidance (n, 52.8%) and AI-based support for strengthening research capacity (n, 51.7%). Science and engineering majors also showed balanced interest across all technologies, with especially high preferences for AI-based information literacy education (n, 35.3%) and research ethics guidance and plagiarism prevention using AI and big data (n, 29.1%). These preferences reflect the high relevance of technology integration in research-intensive, experiment-based fields.

Finally, users’ preferences for specific technologies and services also depended on their occupational background. Healthcare professionals showed strong preferences across all technology types, especially for IoT-based personal research space reservations (n, 43.4%) and VR/AR-based information literacy and usage education (n, 41.2%). Other frequently selected services included cloud-based online guidance, access control, and summarization services, which suggests strong demand for efficient access and use of medical information. Researchers showed evenly distributed preferences, with higher demand for group activity space reservations and external resource integration based on IoT. By contrast, students, educators, and office workers shared a common preference for cloud computing services. Compared to high-level AI services, these groups prioritized effective access to information for learning, education, and daily work, expressing a greater need for practical infrastructure based on cloud environments.

### 4. Factors Influencing Information Use Satisfaction

To address Research Question 4, we performed multiple regression analysis to examine the effects of information and service quality in digital medical information on user satisfaction.

As shown in Table 4, the relationship between the quality of medical information and satisfaction is statistically significant (*p* < .001), with an R² of .588 indicating that 58.8% of the variance in satisfaction could be explained. The adjusted R² was .586. All factors related to information quality significantly influenced satisfaction. Among them, reliability (β = .623) had the greatest impact, followed by recency (β = .380), accuracy (β = .192), and comprehensiveness (β = .145). This indicates that in an environment where users are overwhelmed with information, they assign the highest value to whether the information comes from credible institutions or experts. For medical information users, the timeliness and accuracy of data are critically important for both practice and research.

**Table 4:** Effects of Digital Medical Information and Service Quality on Satisfaction

Variable	Unstandardized coeff.		Standardized coeff.	*t*
	β	SE	β	
**Information quality**				
Constant	.148	.131		1.133**
Recency	.407	.045	.380	8.992***
Accuracy	.205	.043	.192	4.783***
Comprehensiveness	.153	.041	.145	3.774***
Reliability	.667	.035	.623	19.139***
**Service quality**				
Constant	.298	.118		1.133**
Ease of search	.122	.041	.128	2.991**
Website layout	.189	.044	.196	4.335***
Expertise	.090	.043	.089	2.108*
Reliability	.369	.039	.345	9.473***
Personalization	.234	.038	.247	6.118***
Promptness	.273	.044	.282	6.247***

* *p* <.05

** *p* <.01

*** *p* <.001

Similarly, the relationship between service quality and satisfaction is statistically significant (p < .001), with an R² of .628 and an adjusted R² of .624. All service quality variables were found to have a significant impact on satisfaction. The most influential factor was the reliability of the service (β = .345), followed by promptness (β = .282), personalization (β = .247), website layout (β = .196), ease of search (β = .128), and expertise (β = .089). Similar to information quality, users most highly valued the trustworthiness of the service itself. These results indicate that users prefer digital services that are reliable, prompt, and tailored to their individual needs.

## DISCUSSION

This study analyzes the perceptions and actual use of digital medical information and emerging technologies among users of medical science information, with the goal of providing practical evidence for medical libraries to design customized services suited to the digital environment. Based on our research questions, we draw several key implications and practical suggestions from the findings.

First, it is crucial to bridge the gap between the increasingly digital information environment and users' satisfaction levels. Most users reported accessing medical information online, showing a strong preference for scholarly articles, which highlights the need for medical libraries to transition to digital formats. Despite rating the importance of these resources highly, users reported relatively low satisfaction regarding the resources’ usefulness and the accompanying digital services, revealing a clear gap between user expectations and current library offerings. To better compete with external platforms, libraries must refine the user experience. This entails streamlining authentication processes to ensure frictionless access to all resources without repetitive logins [20]. Additionally, libraries should leverage next-generation discovery layers powered by AI. Recent literature highlights the practical benefits of these systems in mitigating information overload and enhancing service efficiency [3, 21]. By applying Natural Language Processing (NLP) and machine learning, such platforms can interpret context-dependent queries and automate evidence synthesis to support clinical decision-making [22]. Implementing such solutions allows for the simultaneous querying of global biomedical databases and local holdings, utilizing AI algorithms to synthesize and rank results effectively without the need for in-house software development.

Second, practical support is necessary to address the disconnect between the growing demand for emerging technologies and users' limited proficiency with them. More specifically, although users expressed significant interest in applying big data and AI to both research and practice settings, anticipating benefits such as information summarization, easier access, and faster information retrieval, their self-rated understanding of and proficiency with digital technologies were relatively low. This indicates that while users perceive these technologies as useful, they struggle with their application. Furthermore, considering the low user awareness regarding copyright and legal regulations, this technical support must be coupled with information ethics education. Such parallel training will ensure that users can navigate the digital landscape and utilize information without legal or ethical complications.

Libraries are encouraged to strategically leverage emerging technologies to enhance the specific dimensions of information and service quality that the IS Success Model identifies as critical drivers of user satisfaction. For instance, the high demand for AI could be addressed by integrating generative AI-powered summarization tools into library services. Such initiatives would align with the goal of enhancing information quality, particularly by targeting recency and perceived reliability—factors found to be significant influences on satisfaction in this study. Similarly, applying big data analytics to library usage logs may enable the development of personalized information curation services. This has the potential to improve service quality by supporting personalization and promptness, two key determinants of user satisfaction identified in our analysis. Finally, cloud computing can provide the essential infrastructure for these services, facilitating the overall system's reliability and accessibility. By mapping specific technological applications directly to the core quality constructs of the IS Success Model, medical libraries can aim to create a user-centered digital ecosystem that effectively bridges the gap between user expectations and satisfaction.

Fourth, user characteristics must be considered when developing differentiated, technology-enhanced services, as our results revealed significant differences in technology preferences and service needs based on users’ gender, education level, academic discipline, and occupation. For instance, respondents in medical-related academic disciplines preferred services integrating big data and AI, whereas those in other majors, such as humanities and social sciences, favored cloud-based e-learning and remote access. Similarly, healthcare workers preferred big data-driven information curation and multimedia services, while the general student group showed greater interest in cloud-based e-learning and electronic resource access. These findings stress the need to move beyond one-size-fits-all service models and implement tailored strategies that reflect the specific needs of distinct user groups.

Fifth, reliability, identified as the most influential factor in user satisfaction, should be the primary priority in both information and service provision, supported by strategies to improve timeliness, accuracy, responsiveness, and personalization. Both information quality and service quality were also found to have significant effects on satisfaction. Reliability showed the strongest association with user satisfaction within both the information quality and service quality dimensions. These results collectively indicate that users value credible information sources and expect services to be personalized and timely. In addition, users reported higher satisfaction with emerging technology-based services that demonstrated strong reliability, accuracy, expertise, and trustworthiness. In particular, users who showed high interest in AI-powered research support services considered reliability the most important factor, followed by accuracy and expertise. Thus, extending beyond traditional collection management, medical librarians must evolve into evaluators of algorithmic reliability. This involves focusing on validating AI-generated outputs, maintaining systematic updates, and developing personalized, data-driven support systems.

Ultimately, these findings highlight the need for medical libraries to further accelerate their evolution into integrated digital ecosystems, building upon their existing technological advancements. By applying the IS Success Model as a theoretical framework, this study identifies information and service quality as key determinants of user satisfaction, while also suggesting that system performance alone is insufficient to meet user needs. In high-stakes fields like medicine, technology-based services must be built on a foundation of human-centric trust and professional expertise to ensure reliability and meaningful user engagement. The variations observed in users’ awareness and satisfaction directly reflect their differing levels of familiarity and readiness toward this technological shift, underscoring the need for strategies that balance advanced implementation with robust educational and professional support.

## LIMITATIONS

This study has several limitations. First, the findings are based on self-reported survey responses, which may reflect users’ subjective judgments or memory rather than actual usage behavior. Therefore, the results may be affected by social desirability bias and recall bias. Second, although this study collaborated with national-level institutions to ensure broad participation, the reliance on voluntary web-based sampling may introduce self-selection bias. Furthermore, as the data is specific to the South Korean medical library and research context, caution is advised when generalizing the findings to other cultural or organizational settings.

Lastly, the use of single-item measures precludes the statistical assessment of internal consistency and may limit the measurement of multi-dimensional constructs. However, to mitigate these limitations and ensure content validity and clarity, the initial questionnaire underwent a pilot review process involving internal researchers and domain experts prior to distribution. Although using single-item measures precludes the assessment of internal consistency reliability, it is considered an appropriate method for measuring unambiguous, narrow concepts [23]. We adopted this approach to minimize respondent burden, consistent with methodologies used in recent technology adoption and service quality research [21, 24–25].

Although this study confirms the influence of information and service quality on satisfaction, it does not account for other potential variables such as users’ research environments, institutional support, or prior educational experiences related to information use. Finally, given the rapidly changing nature of digital technologies, some newly emerging or increasingly relevant technologies may not be adequately reflected due to the timing of the survey.

## SUGGESTIONS FOR FUTURE RESEARCH

To overcome the limitations of self-reporting and accurately capture actual usage, future studies should adopt mixed-methods approaches including log data analysis, observational studies, in-depth interviews, and usability testing. Future research should also investigate the reasons behind the key findings from this study, such as the discrepancy between perceived importance and satisfaction, the gap between perceived usefulness and proficiency in using technology, and users’ low awareness of copyright. Third, to consider the influence of unmeasured external variables, future studies should include personal, institutional, and educational factors—such as research field, digital literacy level, institutional support policies, infrastructure availability, and training experiences—as potential explanatory variables. Fourth, given the rapid pace of technological advancement, longitudinal studies are needed to track changes in user perceptions, technology adoption processes, and the long-term effects of newly implemented services over time. Finally, to ensure the successful implementation and sustainability of technology-based services, further research should examine the perspectives of medical librarians, including their readiness to adopt new technologies, perceptions of role change, competency requirements, training needs, and potential challenges.

## CONCLUSIONS

Our findings confirm that medical information users have a strong desire to access reliable and specialized digital information quickly and conveniently. In this study, users showed significant enthusiasm for the adoption of emerging technologies—particularly AI and big data—for purposes such as information summarization, personalized access, and research support. However, their relatively limited understanding and proficiency regarding these technologies highlight the need for more active education and support from medical libraries.

We also find that user preferences for technologies and services varied according to individual characteristics, with the reliability of both information and services emerging as the most influential factor in user satisfaction. These findings suggest that medical libraries should move beyond the simple adoption of emerging technologies and instead implement user-centered digital information strategies to improve satisfaction and meet actual needs. Specifically, rather than simply expanding collections, libraries should prioritize the seamless integration of AI and data analytics into their existing information services and offer targeted training to bridge the user proficiency gap. In doing so, medical libraries can reinforce their role as central knowledge hubs supporting future medical research, education, and clinical practice. Implementing these recommendations can help medical libraries position themselves as key drivers of efficient and reliable, healthcare information access.

## Data Availability

The data that support the findings of this study are openly available on the Open Science Framework (OSF) at: https://osf.io/3b6jh/files/osfstorage.

## SUPPLEMENTAL FILES

**Appendix A:** Survey Instrument

**Appendix B:** Library Service Codebook
