# Supplementary material for: Perceptions of digital medical information services applying new technologies
Source: J Med Libr Assoc. 2026 Jul 14;114(3):208–21. doi: 10.5195/jmla.2026.2314 (PMC13367307; doi:10.5195/jmla.2026.2314)
Supplement: Supplementary file 1 — Appendix A: Survey Instrument [file jmla-114-3-208-s01.docx]

**Appendix A. Survey Questionnaire**

## General Information

1. What is your gender?

□ Male □ Female □ Prefer not to answer

2. What is your age group?

□ Teen □ 20s □ 30s □ 40s □ 50s □ 60s or older

3. What is your highest level of education?

□ High school graduate □ Junior college graduate □ University graduate □ Graduate school □ Other

4. What is your academic major?

□ Medical-related fields □ Science and Engineering □ Humanities and Social Sciences □ Other

5. What is your occupation?

□ Healthcare service worker □ Researcher □ Educator □ Student □ Office worker □ Other

6. Are you currently working in the health/medical service sector?

□ Yes □ No

7. How many years of work experience do you have?

□ Less than 2 years □ 2–5 years □ 5–10 years □ 10–15 years □ 15–20 years □ 20–25 years □ Over 25 years

**Perceptions of the Use of Medical Information and Technology**

8. Do you primarily access medical information online (web-based) or offline (in-person)?

□ Online □ Offline

9. Which types of medical information resources do you most frequently use? (Select one)

| Types | |
| --- | --- |
| Scholarly articles in medical science | □ |
| Research outputs | □ |
| General health-related information | □ |
| Publications by government agencies or ministries | □ |
| Other | □ |

10. Which platforms in the medical and scientific fields do you most often use for work or research? (Select up to 3)

| Platforms | |
| --- | --- |
| National information agencies  (e.g., National Library of Korea, National Assembly Library) | □ |
| University libraries | □ |
| Access Medicine | □ |
| Clinical Key | □ |
| CINAHL Complete | □ |
| DBpia | □ |
| JCR | □ |
| KERIS | □ |
| KISTI | □ |
| KoreaMed | □ |
| LWW | □ |
| Medicine Complete | □ |
| NIH | □ |
| ProQuest Medical Library | □ |
| ResearchGate | □ |
| Science Direct | □ |
| SpringerLink | □ |
| UpToDate | □ |
| Web of Science | □ |
| Wiley Online Library | □ |
| Video(e.g., Youtube) | □ |
| Other | □ |

11. Rate the importance of medical information resources. (5-point Likert scale)

12. Rate the usefulness of the resources provided by medical libraries. (5-point Likert scale)

13. Rate your satisfaction with digital services and electronic resources. (5-point Likert scale)

14. Rate your awareness of copyright and perceived freedom to use digital resources. (5-point Likert scale)

**Perceptions of Digital Technologies and Services**

15. What advantages do you expect from using services embedded with emerging digital technologies? (Multiple responses allowed)

| Advantages | |
| --- | --- |
| Summarized information | □ |
| Ease of access | □ |
| Prompt information acquisition | □ |
| Personalized information | □ |
| Ease of tracking current trends | □ |
| Trust in the acquired information | □ |

16. Which educational programs would you like medical libraries to offer? (Multiple responses allowed)

| Programs | |
| --- | --- |
| Guide to collection and database usage | □ |
| library guide | □ |
| How to Use Analytical Tools and Research Assistance Tools | □ |
| Information on how to search for medical science information | □ |
| Guide to the latest AI utilization technology | □ |
| Other | □ |

17. Rate the perceived usefulness of digital technologies. (5-point Likert scale)

18. Rate your understanding of digital technologies. (5-point Likert scale)

19. Rate your proficiency in using digital technologies. (5-point Likert scale)

20. Which digital technologies would you like to apply in research and clinical settings? (Select one)

| Digital technologies | |
| --- | --- |
| Big data | □ |
| Artificial intelligence | □ |
| Cloud computing | □ |
| Next-generation/mobile communication | □ |
| Virtual/augmented reality |  |
| Other |  |

21. For each technology type below, please select the library services where you hope to see these technologies integrated. (Multiple responses allowed)

| Technologies-Service | | |
| --- | --- | --- |
| Virtual/augmented reality | |  |
|  | New technology/creative education and space provision | □ |
|  | Information literacy/user education | □ |
| Big data | |  |
|  | Trend/network analysis | □ |
|  | Multimedia resource provision/guidance | □ |
|  | Provision/use of library holdings | □ |
|  | Research capacity enhancement support | □ |
|  | Online service provision/guidance | □ |
|  | E-learning provision/integration | □ |
|  | Information literacy/user education | □ |
|  | Knowledge & information curation | □ |
|  | Academic journal information guidance | □ |
| **Artificial intelligence** | |  |
|  | Research capacity enhancement support | □ |
|  | Research ethics guidance/plagiarism prevention | □ |
|  | Online service provision/guidance | □ |
|  | Summarization service | □ |
|  | Information literacy/user education | □ |
| **Next-generation/mobile communication/IoT** | |  |
|  | Personal research space reservation | □ |
|  | Group activity space reservation | □ |
|  | Multimedia resource provision/guidance | □ |
|  | Center access control | □ |
|  | Provision/use of library holdings | □ |
|  | New technology/creative education and space provision | □ |
|  | Online service provision/guidance | □ |
|  | External resource provision/integration | □ |
|  | Remote access to electronic resources | □ |
|  | E-learning provision/integration | □ |
|  | Information literacy/user education | □ |
|  | Knowledge & information curation | □ |
| **Cloud computing** | |  |
|  | Online service provision/guidance | □ |
|  | Remote access to electronic resources | □ |
|  | E-learning provision/integration | □ |
| **Other** | | □ |

22. information quality (5-point Likert scale)

- The information is up‑to‑date.
- The information is accurate.
- The information is comprehensive.
- The information is reliable.

23. Service quality (5-point Likert scale)

- The service is easy to search and navigate.
- The website layout is clear and user‑friendly.
- The service is reliable.
- The service is prompt.
- The service is personalized to my needs.
- The service demonstrates professional expertise

24. Satisfaction (5-point Likert scale)

- The medical information helped me complete tasks promptly.
- The information improved my work performance.
- The information was easy to access and use.
- The information was useful for my needs.
- Overall, I am satisfied with the medical information I accessed.
