# Supplementary material for: Perceptions of digital medical information services applying new technologies
Source: J Med Libr Assoc. 2026 Jul 14;114(3):208–21. doi: 10.5195/jmla.2026.2314 (PMC13367307; doi:10.5195/jmla.2026.2314)
Supplement: Supplementary file 2 — Appendix B: Library Service Codebook [file jmla-114-3-208-s02.docx]

**Appendix B. Library Service Codebook**

| **Service** | **Definition** | **Examples** |
| --- | --- | --- |
| Academic Journal Information Guidance | Support for accessing, evaluating, and using academic journals effectively. | Explaining journal rankings, how to use Scopus/SCI, and finding relevant journals. |
| Center Access Control | Systems for controlling and managing access to library or research center spaces. | Access card systems, smart door locks. |
| Cloud-based Access to Electronic Resources | Support for accessing electronic resources from outside the campus via cloud or remote services. | Using EZproxy, VPN connection instructions. |
| E-learning Provision/Integration | Provision or integration of online learning systems and content with learning platforms. | Integration with Moodle, offering e-learning contents. |
| External Resource Provision/Integration | Services that integrate academic resources from external platforms. | Access to JSTOR, PubMed, or ProQuest. |
| Group Activity Space Reservation | Reservation services for collaborative or group workspaces. | Booking small discussion rooms or meeting rooms. |
| Information Literacy/User Education | Programs designed to improve users' ability to locate, evaluate, and use information effectively. | Library orientation, citation tool workshops, search strategies tutorials. |
| Knowledge & Information Curation | Curation and delivery of trusted and relevant information based on user or subject needs. | Subject guides, recommended reading lists, pathfinders. |
| Multimedia Resource Provision/Guidance | Support for accessing and utilizing multimedia and non-print materials. | Streaming video, audiobooks, multimedia databases. |
| Online Service Provision/Guidance | Guidance and access to various online library services. | Chat reference, online manuals, virtual helpdesk. |
| Personal Research Space Reservation | Reservation service for individual study or research spaces. | Quiet study rooms, carrels, soundproof booths. |
| Provision/Use of Library Holdings | Support for accessing and utilizing printed and electronic library materials. | Book borrowing, reserve materials, library catalog search. |
| Remote Access to Electronic Resources | Technical and instructional support for remote access to subscribed resources. | Proxy settings guides, remote login instructions. |
| Research Capacity Enhancement Support | Programs and services designed to improve users’ academic research skills. | Workshops on research methodology, academic writing consultations. |
| Research Ethics Guidance/Plagiarism Prevention | Education and tools to promote academic integrity and ethical research practices. | Plagiarism detection tools, research ethics workshops. |
| Summarization Service | Providing summaries of lengthy documents or data to support users' understanding. | Abstract writing, summarizing core contents of reports or articles. |
| Technology-Integrated Creative Learning Spaces | Support for learning and research using new technologies and creative spaces. | Makerspaces, VR/AR labs, 3D printing studios. |
| Trend/Network Analysis | Support for analyzing research trends and visualizing knowledge networks. | Citation analysis, topic clustering, co-authorship network visualization. |
